# Infectious Mononucleosis: A Case Report With Unusual Features and Abnormal Laboratory Findings

**DOI:** 10.7759/cureus.14790

**Published:** 2021-05-01

**Authors:** Ammar Alli, Farah Nabil, Juan Fernando Ortiz

**Affiliations:** 1 Internal Medicine, Tishreen University Faculty of Medicine, Latakia, SYR; 2 Internal Medicine, Universitat de Barcelona, Barcelona, ESP; 3 Faculty of Health Sciences, University of Zaragoza, Zaragoza, ESP; 4 Neurology, Universidad San Francisco de Quito, Quito, ECU; 5 Neurology, Larkin Community Hospital, Miami, USA

**Keywords:** infectious mononucleosis, epstein–barr virus

## Abstract

Despite the widespread Epstein-Barr virus (EBV) infections, we continue to see new reports with strange and unusual manifestations of the infection, which raises the question of how well we understand this pathogen. The age of contracting the infection is increasing in developed countries, which is changing the clinical presentation of those who get infected during their adolescence or early adulthood. In these stages, liver involvement is more prominent, and other physical symptoms are less apparent. Therefore, an update on infectious mononucleosis (IM) variable manifestation is required to make healthcare providers aware of this shift. This case stands as an example of the new shift as a patient scheduled for elective surgery suddenly presented with subclinical hepatitis caused by primary EBV infection. Our patient presented with few physical symptoms but had a classical picture of EBV-induced hepatitis on blood analysis. The diagnosis was missed by many physicians due to the varied presentations of IM. This case corresponds to the new evidence that suggests that hepatic involvement is one of the most prominent manifestation in the adult population with primary EBV infection.

## Introduction

Epstein-Barr virus (EBV) is a member of the herpes family and one of the most common viruses known to infect humans. It is estimated that 90% of the general population becomes infected with the virus before the age of 30 [1]. However, in most cases, the infection is self-limiting and rarely leads to dangerous complications; fewer than 1% of cases experience serious complications [1].

EBV infection ranges from asymptomatic disease to a spectrum of illnesses. Similar to most viral infections, children are usually asymptomatic, while young adults show the classic symptoms of infectious mononucleosis (IM). Patients older than 60 years frequently present with jaundice, minor lymphadenopathy, sore throat, and splenomegaly [2].

Liver involvement is seen in 80-90% of IM cases. It is mainly characterized by a transitory elevation of liver enzymes [3]. The self-limiting and benign nature of the infection hinder healthcare providers’ ability to diagnose certain primary EBV cases and answer patient’s question regarding the infection course. Here, we present an unconventional IM case with an atypical presentation and an unusual liver laboratory pattern, which differs from the traditional patterns of the infection.

This case aims to bring healthcare providers’ attention to the different manifestations of IM in young individuals compared to adolescents as the disease mainly presents as subclinical hepatitis with no other symptoms.

## Case presentation

A 29-year-old Syrian man called his primary care physician on day one before his scheduled elective surgery on day six. He initially complained of having “white spots” on his right tonsil. The General Practitioner (GP) prescribed him antibiotics immediately, assuming he had streptococcal pharyngitis.

On the second day, the patient came to the clinic for further evaluation. On physical examination, the patient had two inflamed lymph nodes below the jaw and an extensive membrane covering the right tonsil. His temperature was 37.0°C. He was prescribed intramuscular ceftriaxone twice a day along with 2 g of oral amoxicillin per day. His physical examination was otherwise normal. He reported no fever, malaise, or any sore throat. He reported that he sought medical help only because he had a scheduled surgery and wanted to make sure everything was alright.

On the fourth day, the patient was referred to an otolaryngologist for further evaluation. His left tonsil started showing increasing numbers of white spots as well. No improvement was seen on the tonsils. The otolaryngologist repeated the physical and confirmed the findings of the GP, and instructed the patient to continue with the antibiotics.

On the fifth day, the patient visited the infectious disease department in a local hospital, where the resident doctor instructed him to stop the medications and order some lab work for him.

On the sixth day, the lab results came back confirming a diagnosis of IM. The patient was asked to follow-up with the infectious disease department weekly to monitor his condition. Table 1 shows the first laboratory results of the patient.

**Table 1:** Laboratory results of the patients. ALP: alkaline phosphatase; ALT: alanine transaminase; ASLO: anti-streptolysin O; AST: asparate transaminase; CRP: C-reactive protein

Blood analysis first week	Patient’s results	Normal range
While blood cells	10,800 (62% lymphocytes)	4,500-10,500/mm^3^
ASLO	29	Up to 200 IU/mL
CRP	8.9	Up to 6 mg/L
ALT	370	Up to 41 U/L
AST	188	Up to 37 U/L
ALP	181	40-129 U/L
Monospot test	Positive	Negative

Table 2 shows the second blood analysis of the patient.

**Table 2:** Laboratory results of the patient. ALP: alkaline phosphatase; ALT: alanine transaminase; AST: asparate transaminase; CRP: C-reactive protein

Blood analysis second week	Patient’s results	Normal range
White blood cells	7,300 (59% lymphocytes)	4,500-10,500/mm^3^
CRP	2.3	Up to 6 mg/L
ALT	417	Up to 41 U/L
AST	108	Up to 37 U/L
ALP	184	40-129 U/L

Table 3 shows the third blood analysis of the patient.

**Table 3:** Laboratory results of the patient. ALP: alkaline phosphatase; ALT: alanine transaminase; AST: asparate transaminase; Gamma GT: gamma glutamyltranspeptidase

Blood analysis third week	Patient’s results	Normal range
White blood cells	7,900 (54% lymphocytes)	4,500-10,500/mm^3^
ALT	154	Up to 41 U/L
AST	50	Up to 37 U/L
ALP	141	40-129 U/L
Gamma GT	71	11-51 U/L

Table 4 shows the fourth blood analysis of the patient.

**Table 4:** Laboratory results of the patient. ALP: alkaline phosphatase; ALT: alanine transaminase; AST: asparate transaminase; Gamma GT: gamma glutamyltranspeptidase

Blood analysis fourth week	Patient’s results	Normal range
White blood cells	7,700 (37% lymphocytes)	4,500-10,500/mm^3^
ALT	81	Up to 41 U/L
AST	16	Up to 37 U/L
ALP	114	40-129 U/L
Gamma GT	51	11-51 U/L

## Discussion

EBV is one of the most common viruses that are known to cause or be associated with a variety of human diseases. Understanding primary EBV infection with its variable range of manifestations is crucial to diagnose patients, better evaluate the infection course, and provide better care. On physical examination, only two classical symptoms were present in our patient: two enlarged cervical lymph nodes and pseudomembranous tonsillitis. Therefore, many physicians missed the initial diagnosis. Nevertheless, blood work showed an extensive picture of EBV with one of its most common manifestations, transient mild hepatitis.

Diagnosis and features of the infection compared to our case

In the literature, the most common symptoms of IM include sore throat (94%), cervical lymphadenopathy (81%), fatigue (72%), and upper respiratory symptoms (64%) [1]. The clinical triad of fever, sore throat, and lymphadenopathy is considered an indicator for primary EBV infection.

A study reported that patients with IM developed a rash at a rate of almost 100% after receiving penicillin which was explained as a period of transient hypersensitivity toward penicillin during the course of the infection [4,5]. The incidence of different lab results varied between studies.

In our case, the patient presented with two enlarged cervical lymph nodes and a pseudomembrane on his tonsils. Other classical IM symptoms were not present. His physical examination and vital signs were within normal limits. After receiving aggressive treatment with PO amoxicillin 2 g/day and intramuscular ceftriaxone 2 g/day for four days, the patient did not develop any rash, even though most IM patients develop a rash after penicillin therapy.

The patient was diagnosed after his Monospot test came positive. Studies show that 85-90% of young people test positive on Monospot test during the course of EBV infection [6], securing an accuracy rate of 71% to 90% for the diagnosis of IM [2]. The patient’s peripheral blood smear showed more than 10% of atypical lymphocytes. The elevated transaminases and lymphocytosis were helpful in confirming the diagnosis.

Liver involvement

In primary EBV, liver involvement is seen in more than 80% of cases [3]. Therefore, it is important to understand this aspect of the disease. Liver enzymes including alanine aminotransferase (ALT), aspartate aminotransferase (AST), alkaline phosphatase (ALP), and gamma glutamyltranspeptidase (gamma GT) are elevated. The predominant type is a transient cholestatic (conjugated hyperbilirubinemia, elevated ALP, and gamma GT) liver disease seen in 59% of cases [3].

**Table 5:** Comparison of expected liver enzymes and liver enzymes of our patient. ALT: alanine aminotransferase; AST: aspartate aminotransferase; EBV: Epstein-Barr virus

Liver enzymes	Our patient	Expected increase due to EBV
ALT	370-417 U/L	Up to 205 U/L
AST	188-108 U/L	Up to185 U/L

According to recent studies, EBV hepatitis frequency increases with age [7]. Thus, healthcare providers should consider EBV as a possible cause of hepatitis where mild elevation of transaminases is reported. During EBV, hepatitis elevation of transaminase is usually mild; in other words, it is less than five-fold of the normal levels [3]. As seen in our patient, ALT elevation of more than five-fold is less common and very unusual.

EBV hepatitis is benign and self-limiting. However, a proper understanding of its course is beneficial to educate healthcare providers when they encounter surprising laboratory results and answer patients’ questions about the infection pattern.

A study that included 47 primary EBV patients showed that the rise of ALT peaked three times on days 8, 11, and 13 (during the second week); AST peaked two times on days 8 and 11 (also during the second week); ALP peaked randomly and remained elevated even after 30 days, and gamma GT peaked one time after 15 days [3]. Our patient had an increase in ALT levels in the second week, consistent with the normal variations and peaks seen in other studies. It is important to know that these variations are normal, carry no clinical significance, and are not correlated with the changes to other findings such as lymphocytosis [3].

After his second blood analysis, our patient felt anxious as he had an increased ALT (370 increased to 417 U/L). Therefore, he was instructed to follow a low-fat diet rich in carbohydrates. According to the literature, these variations were normal and did not require following restrictive diets. Nevertheless, this approach was considered a precarious measure.

## Conclusions

In conclusion, despite the financial and technical difficulties that healthcare providers and patients face currently in Syria, this case is unique as it shows few clinical findings consistent with primary EBV. However, it documents an important manifestation of EBV-induced hepatitis in a young, healthy adult male. This study sheds light on the importance of recognizing primary EBV in older populations and taking into account the possible decrease in classic IM symptoms and the increasing likelihood of subclinical hepatitis.
